# Supplementary figures and images for: Lycium barbarum Polysaccharide Regulates the Lipid Metabolism and Alters Gut Microbiota in High-Fat Diet Induced Obese Mice
Source: Int J Environ Res Public Health. 2022 Sep 24;19(19):12093. doi: 10.3390/ijerph191912093 (PMC9566073; doi:10.3390/ijerph191912093)

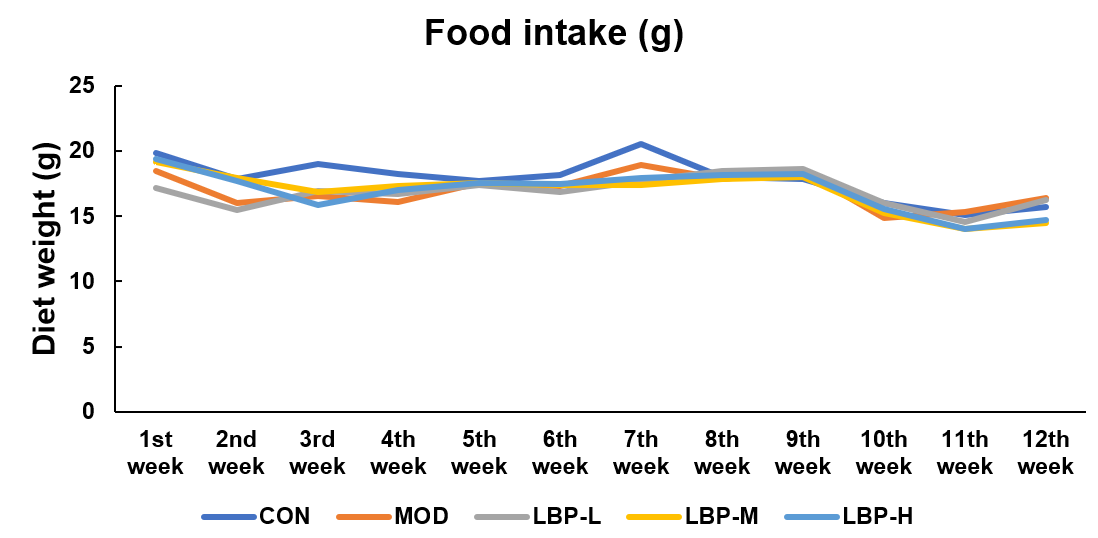

Supplement: Supplementary file 1 [file ijerph-19-12093-s001.zip › food consumption.png]
